# FitForLife: study protocol for a randomized controlled trial

**DOI:** 10.1186/s13063-015-1071-9

**Published:** 2015-12-04

**Authors:** Yvonne Forsell, Mats Hallgren, Maria Mattson, Orjan Ekblom, Catharina Lavebratt

**Affiliations:** Division of Epidemiology and Public Health Intervention Research (EPHIR), Department of Public Health Sciences, Karolinska Institutet, Solna, 171 77 Sweden; Midhagen Psychiatric Outpatient Clinic, Sankt Göransgatan 126, Stockholm, 112 45 Sweden; The Swedish School of Sport and Health Sciences, Box 5626, Stockholm, 114 86 Sweden; Department of Molecular Medicine and Surgery, Karolinska Institutet, Solna, 171 77 Sweden

**Keywords:** Psychosis, Treatment, Exercise, Intervention

## Abstract

**Background:**

Psychosis is a serious mental illness that typically emerges during early adulthood. The disorder is characterized by inactivity, cognitive deficits and the need for ongoing support. Regular exercise has mood enhancing and anxiolytic effects that could benefit this patient group. To date, few studies have examined the effects of prescribed exercise on autonomy, health and cognitive functioning in psychosis.

**Methods/Design:**

This is a single-center, randomized controlled trial (RCT) with a 3-month follow-up. Usual care plus a 12-week supervised exercise program will be compared to usual outpatient care alone. The primary outcome will be patient autonomy measured by the Camberwell Assessment of Need (CAN) schedule – clinician rated. Secondary outcomes include cardiovascular risk factors, cognitive functioning, substance abuse, body awareness, depression and mood state. Changes in inflammatory markers and microbiotica will be explored. The feasibility of using patients as exercise trainers will also be assessed.

**Discussion:**

The treatment potential for exercise in psychosis is large because most individuals with the disorder are young and inactive. The study is one of the first to comprehensively assess the effects of regular exercise in young adults with psychosis. Sessions will be closely supervised and adjusted to meet patient needs. Both the feasibility and treatment effects of exercise interventions in psychosis will be discussed.

**Trial registration:**

German Clinical Trials Register DRKS00008991 7 August 2015.

## Background

Psychosis is a serious mental disorder characterized by a loss of contact with reality and deterioration in social and occupational functioning. The most serious form of the disorder, schizophrenia, has a lifetime prevalence of 5.5 (SD = 4.5) in 1,000 persons [1]. The mean age of onset is 18 years in males and 25 in females [2]. Schizophrenia is associated with elevated suicide rates and an increased risk of premature death related to a range of comorbid somatic conditions [3]. Rates of depression vary between 25–81 % [4], and substance abuse is highly prevalent. Cognitive impairment is a core feature of schizophrenia, which can adversely affect occupational functioning and life quality [5]. Deficits typically appear in attention, declarative memory and higher-order problem solving [6]. The speed of processing, working and long-term memory can also be affected, as are abilities that are essential to school and working life. Several studies have proposed that neuroinflammation and autoimmunity are involved in the etiology and pathogenesis of schizophrenia [7, 8]. The stress-vulnerability model suggests that lowering biological vulnerability and reducing stress may help prevent relapse. Reducing biological vulnerability with medication remains a cornerstone of treatment. Increasingly, psychosocial interventions have also focusing on resilience to stress [9].

Inactivity is another key feature of psychosis; most individuals with the disorder do not meet the minimum physical activity levels recommended for general health [10]. This is problematic because regular exercise has positive neurobiological effects, which are likely to benefit this patient group. For example, exercise affects the hypothalamic-pituitary-adrenal (HPA) axis, which in turn modulates stress-reactivity [11, 12]. Exercise also influences hippocampal cell proliferation [13], and serotonergic release [14], producing an anti-depressant effect when performed regularly. Regular physical activity has been shown to lower systemic inflammation [15], which may be of importance in psychotic patients. According to three Cochrane reviews, physical exercise has moderate effects on depression equivalent to anti-depressant treatment [16–18]. Other beneficial effects include improved sleep quality [19] and increased neural growth factor expression, which affects neurogenesis, learning and memory [20]. Recent studies suggest that exercise may also reduce cravings for alcohol and other drugs in substance dependent individuals [21, 22]. Despite this evidence, few studies have explored the effects of exercise in patients with psychosis, a physically inactive patient group with high rates of comorbid depression and substance abuse.

The primary objective of FitForLife is to examine the effects of a 12-week physical exercise program on autonomy in young adults with psychosis. Secondary aims are to explore changes in general health, cognitive ability, substance use, body awareness, depression and mood states. Blood and feces samples will be requested to assess changes in inflammatory markers and microbiotica. The feasibility of educating patients as exercise trainers will also be assessed. We hypothesize that the intervention will lead to improvements in all outcomes assessed.

## Specific research questions

Specific research questions are as follows:

1. Can a supervised exercise intervention for psychosis improve patient autonomy (that is, their need for care and assistance)?
2. What are the effects of regular exercise on cardiovascular risk factors (body weight, waist and hip circumference, blood pressure, blood lipids), maximal oxygen uptake (VO_2 max_), and inflammatory markers?
3. Can regular exercise improve cognitive functioning and body awareness in psychosis?
4. Is regular exercise associated with reduced comorbidity, specifically, depression, anxiety and substance abuse?
5. What are the effects of regular exercise on symptom recurrence and hospitalization?
6. Does regular exercise improve the microbiotic profile of patients, and are these changes correlated with other health-related outcomes?
7. What are the short-term (acute) effects of exercise on self-rated mood in psychosis?
8. Is it feasible to train psychotic patients as exercise trainers and role models?

## Method/Design

### Setting

All patients will be recruited via the ‘Midhagen’ outpatient unit for young adults (aged 18 to 45 years) with first-episode psychosis. Located in central Stockholm, the clinic delivers need-based care with the aim of maximizing patient autonomy and life quality. Various interventions are offered, including counselling-support, medication and education. Clinical staff are specialized in psychiatry, clinical psychology or psychiatric nursing. Approximately 200 patients are registered at the clinic each year, and the majority has schizophrenia. A minority have schizoaffective disorder or drug-induced psychosis. If the number of patients willing to participate is insufficient, the study will extend to a second psychiatric outpatient clinic located about 20 kilometers outside Stockholm.

### Study design, randomization and blinding

**Fig. 1:**
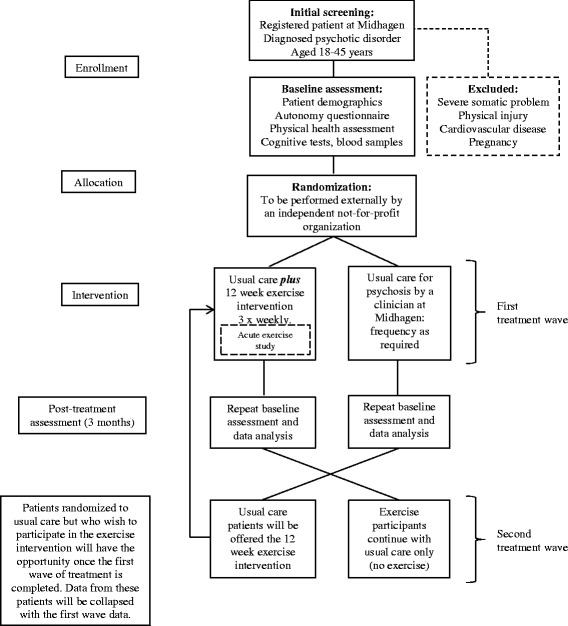
CONSORT diagram showing the proposed flow of participants through FitForLife. FitForLife is a randomized controlled trial (RCT) comparing the effects of standard outpatient treatment for psychosis to standard care plus a 12-week supported exercise intervention. The trial includes an embedded study of the acute effects of exercise on mood states

Single-center, two-group parallel, randomized controlled trial (RCT) with a 3-month post-treatment assessment (study end-point). Random allocation of the patients will be performed externally. A psychiatric nurse and research-assistant will be trained to conduct patient assessments before and after treatment. Assessors will, in most instances, be aware of the patient’s treatment allocation. Genuine blinding of assessors is not feasible given the high degree of research assistant involvement in the project as a whole. It is also likely that many patients will discuss their treatment will the assessors. Patients randomized to the exercise intervention will also receive usual care for psychosis administered by their clinician. The comparison group in this study will be an active (wait-list) control group that will receive need-based care at the clinic only. This will normally consist of medication and counseling/supportive therapy as required. Changes on key outcomes will be compared before and after treatment within and between the two allocated groups. All patients in the control condition will subsequently be offered the exercise intervention after the first group have completed treatment; that is, 12 weeks after the baseline assessment. This second round of exercise sessions will be led by patients with psychosis who have been educated as trainers (see description below). The feasibility of using patients as trainers will be assessed through a qualitative survey conducted at the end of the study and reported separately. The flow of participants through the trial is shown in Fig. 1.

### Affective responses to exercise

To assess the affective response of patients to acute (short-term) bouts of exercise, a separate study will be embedded within the RCT design. Patients will complete the Positive and Negative Affect Scale (PANAS) [23] 5 to 10 minutes before and after each exercise session. Changes in mood states over time will be examined and reported. Affective responses to acute bouts of exercise have been shown to predict rates of exercise adherence, and are therefore relevant to measure. Specifically, positive mood responses during and following exercise are associated with higher rates of future exercise adherence, whereas negative affective responses have been linked to lower rates of adherence [24].

### Ethics

The study has received ethical approval by the Regional Ethics Committee (Regionala Etikprovninsnämnden, EPN), Stockholm, number 2015/808-31/2. The trial is registered with German Clinical Trials Register: DRKS00008991. Informed consent will be obtained from all participants prior to inclusion. All patients must agree to participate voluntarily and will be free to withdraw from the study at any time. Patients randomized to usual care who express interest in receiving the exercise intervention will be given the opportunity to participate in the exercise treatment after the first wave of treatment is completed.

### Participants

The study will include 80 patients: 40 will receive the exercise intervention, and 40 will serve as active (wait-list) controls. Both new and existing patients will be invited to participate.

Inclusion criteria: All patients attending the clinic with a diagnosed first-episode psychotic disorder who provide informed consent and wish to participate will be included.

Exclusion criteria: Patients with severe somatic disorders or injuries that make it difficult or unsafe to exercise will be excluded. Patients with a history of cardiovascular disease and pregnant women will also be excluded.

### Screening interview

A research assistant or nurse will initially perform a brief screening interview at the clinic to determine patient eligibility using the criteria outlines above. Suitable patients will then be invited to participate in the trial, informed consent obtained, and a baseline assessment conducted prior to randomization.

### Baseline and post-treatment assessments

Assessments will include the following domains/measures:

1. Demographics will be assessed, including age, gender, occupation, sick leave (baseline only; taken from patient files).
2. Diagnosis, age of symptom onset, number of psychosis-related hospital admissions, medication use (also from patient files, where available) will be assessed.
3. Autonomy will be assessed by the Camberwell Assessment of Need (CAN) – clinician rated questionnaire [25]. CAN is a validated instrument used to understand the health and social needs of adults with severe mental health problems. It covers 22 domains of an individual’s life, such as accommodation, self-care, daytime activities, psychological distress, physical health and relationships.
4. Depression severity will be measured using the Montgomery-Åsberg Depression Rating Scale (MADRS) [26].
5. Habitual physical activity levels ‘in a typical week’ (frequency and intensity) will be assessed using a questionnaire developed by the Swedish Institute of Sport and Health Science (GIH:Gymnastik- och idrottshögskolan) and previously used in a nationwide lifestyle study [27].
6. Fitness will be assessed using a modified Åstrand VO2-submax cycle ergometer test [28].
7. Height and weight will be assessed to estimate body mass index (BMI), waist and hip circumference and blood pressure.
8. Body Awareness will be assessed by the Body Awareness Scale [29].
9. Cognitive ability will be assessed by the CogState Brief Battery [30], Trailmaking [31] and Stroop tests [32].
10. Blood and stool samples will be taken to test fatty acids, inflammatory markers (for example, IL-10, IL-1ra and TNF-alpha) and microbiotic profile (samples to be assessed within the Institute for Molecular Medicine and Surgery, Karolinska Institutet).

### Exercise intervention

Exercise sessions will be designed and supervised by exercise science graduates from the Swedish School of Health and Sports Medicine (GIH), under the supervision of an experienced exercise physiologist. GIH has extensive experience developing training protocols for patients with various health conditions. The graduates will also educate and support the six patients who volunteer to work as exercise trainers during the second phase of the study. Exercise sessions will be available five times/week during the 12-week intervention, and participants will be encouraged to attend three sessions each week. The training sessions will consist of a 5 to 10 minute warm-up, 30 minutes of aerobic training, 15 minutes of body-weight resistance training, and a 5 to 10 minute cool-down (about 1 hour in total). Participants will be encouraged to train at a level appropriate to their current fitness and ability, with the aim of reaching a moderate intensity (that is, three times or more higher that resting metabolic rate). Intensity and total training volume will be estimated from the accelerometer data and used in the analyses. To maximize participation and adherence, we aim to offer exercise classes that appeal to the participants, with varying types of exercises and shifting between indoor and outdoor activities. However, most sessions will be conducted indoors; outdoor sessions are dependent on weather conditions. Regular meetings will be scheduled with the trainers and participants to discuss the classes, and there will be capacity to adjust the activities. Six patient (three males, three females) will be invited to become exercise trainers for the second round of patients. Exercise science students from GIH will train these patients. The selection of patient-trainers will be made with clinical staff; six patients will be paid to lead regular exercise classes. The intention is that the trainers will serve as role models to increase participant empowerment.

### Objective assessment of physical activity

All patients randomized to the exercise group will be provided a hip-worn accelerometer (‘FitBit Zip’ device) to objectively measure and record daily physical activity levels, including the total number of steps taken and minutes/hours spent in light, moderate and vigorous activity. The device enables patients to set activity goals and receive daily feedback. It can be used with Smartphones and computers, and it is possible to store data from multiple devices centrally. This will be achieved using the online ‘Fitabase’ program.

### Statistical analysis

Participant characteristics, stratified by treatment group (exercise versus active control) will be presented. Baseline differences will be tested using t-tests for continuous variables and Chi-square tests for categorical outcomes. Parametric test assumptions will be examined and reported. Nonparametric data will be transformed or alternatively analyzed using nonparametric statistical tests. Multiple imputation will be used to replace missing internal values where appropriate. Within-group changes in the primary outcome (autonomy) and secondary outcomes (health, cognitive ability, substance use, etcetera) will be analyzed using paired sample t-tests. To address the primary research question, intention-to-treat analyses will be performed. The effect of group allocation on autonomy and all secondary outcomes at 3 month follow-up will be assessed using linear regression models (logistic for binary outcomes), using both the mean difference (baseline to 3 months) and total scores as dependent variables. Where the total score is used as the outcome, baseline group differences in covariates will be adjusted. Data from individual accelerometers will be pooled to objectively assess changes in physical activity levels before and after treatment. All analyses will be performed using SPSS 22.0. All tests will be two-sided and *P*-values of less than 0.05 will be considered statistically significant.

## Discussion

A Cochrane review from 2010 concluded that exercise interventions for schizophrenia are feasible and may have beneficial psychobiological effects [33]. However, only three studies fulfilled the review inclusion criteria with small sample sizes of 12, 13 and 61 patients, respectively. A recent systematic review and meta-analysis including eight studies showed only a modest increase in levels of exercise and no changes in symptoms or body mass index following exercise [34]. However, the studies varied widely in exercise treatments, sample size, age-range and outcomes measured. Only two studies used a validated measure of exercise as the primary outcome, the 6-minute walking test. Moreover, exercise intensity was not measured; thus, benefits of the interventions were difficult to compare [34]. The effects of exercise on cognition in psychosis have rarely been studied. One study reported a 34 percent improvement in short-term memory, but included only eight patients and eight controls [35].

The treatment potential for exercise in psychosis is large because most individuals with the disorder are sedentary and inactive. This study will be one of the first to comprehensively assess the health-related effects of regular exercise in young adults with psychosis. Exercise interventions for this patient group need to be carefully adjusted and supervised; motor coordination and sensory integration may be impaired, which can adversely affect coordination and body awareness [36]. Supervision of exercise programs has been shown to increase adherence and improve outcomes in depression studies [37]. For these reasons, the current study will involve closely supervised, tailored exercise sessions that will be adjusted to meet patient needs. The objective assessment of physical activity is another important design consideration, as memory and recall may be impaired. Some novice exercisers could be unfamiliar with the sensation of aerobic activity, and may interpret these feelings as distress or anxiety. Care will be taken to ensure that novice exercisers begin with low-intensity exercises so they have time to become familiar with the physical and affective changes often associated with exercise [38]. All classes will be supervised by qualified exercise-trainers, and a psychiatric nurse will be readily available should patients feel distressed or require assistance. While psychosocial interventions in psychosis have expanded rapidly in recent years, exercise interventions have received less attention. Results will be used to inform clinicians and researchers about the feasibility and effects of exercise as an adjunct treatment for psychosis.

### Trial status

The trial is due to commence in September 2015.

## Abbreviations

BMI: Body Mass Index
CAN: Camberwell Assessment of Need
Gymnastik- och idrottshögskolan - GIH: Swedish Institute of Sport and Health Science
HPA: Hypothalamic-Pituitary-Adrenal axis
IL: Interleukin
MADRS: Montgomery-Åsberg Depression Rating Scale
PANAS: Positive and Negative Affect Scale
RCT: Randomized Controlled Trial
Regionala Etikprovninsnämnden - EPN: Regional Ethics Committee
SPSS: Statistical Package for the Social Sciences
TNF- α: Tumor Necrosis Factor-alpha
VO_2 max_: Maximal oxygen uptake
